# The Unexpected Holiday Souvenir: The Public Health Risk to UK Travellers from Ticks Acquired Overseas

**DOI:** 10.3390/ijerph17217957

**Published:** 2020-10-29

**Authors:** Emma L. Gillingham, Benjamin Cull, Maaike E. Pietzsch, L. Paul Phipps, Jolyon M. Medlock, Kayleigh Hansford

**Affiliations:** 1 Medical Entomology and Zoonoses Ecology, Emergency Response Department, Public Health England, Porton Down, Salisbury SP4 0JG, UK; cull0122@umn.edu (B.C.); Maaike.Pietzsch@phe.gov.uk (M.E.P.); Jolyon.Medlock@phe.gov.uk (J.M.M.); kayleigh.hansford@phe.gov.uk (K.H.); 2 Wildlife Zoonoses and Vector-Borne Research Group, Department of Virology, Animal and Plant Health Agency, Addlestone, Surrey KT15 3NB, UK; Paul.Phipps@apha.gov.uk

**Keywords:** tick-borne pathogens, *Ixodes ricinus*, *Amblyomma americanum*, *Dermacentor*, *Hyalomma*, *Rhipicephalus*, *Ixodes*, *Amblyomma*

## Abstract

Overseas travel to regions where ticks are found can increase travellers’ exposure to ticks and pathogens that may be unfamiliar to medical professionals in their home countries. Previous studies have detailed non-native tick species removed from recently returned travellers, occasionally leading to travel-associated human cases of exotic tick-borne disease. There are 20 species of tick endemic to the UK, yet UK travellers can be exposed to many other non-native species whilst overseas. Here, we report ticks received by Public Health England’s Tick Surveillance Scheme from humans with recent travel history between January 2006 and December 2018. Altogether, 16 tick species were received from people who had recently travelled overseas. Confirmed imports (acquired outside of the UK) were received from people who recently travelled to 22 countries. Possible imports (acquired abroad or within the UK) were received from people who had recently travelled to eight European countries. Species-specific literature reviews highlighted nine of the sixteen tick species are known to vector at least one tick-borne pathogen to humans in the country of acquisition, suggesting travellers exposed to ticks may be at risk of being bitten by a species that is a known vector, with implications for novel tick-borne disease transmission to travellers.

## 1. Introduction

Since the 1950s, there has been year-on-year increases in worldwide international tourist arrivals [1]. In 2017 alone, there were 1,323 million worldwide tourist arrivals, increasing by 84 million international arrivals than 2016 [1]. Similar tourism increases have been seen in the United Kingdom (UK): overseas resident arrivals increased by 4% in 2017 compared with 2016, and 3% more overseas visits were made by UK residents in the same time period [2]. Such frequent movement could increase exposure of travellers to ticks and their pathogens. As ticks are the second most common vectors of disease-causing pathogens in humans [3] such exposure could present unique public health challenges, as ticks from one region may transmit pathogens that are unfamiliar to medical professionals in other parts of the world [4].

To date, several reports have detailed tick detection and removal on recently returned travellers, and ticks acquired in Africa, Asia, Australia, North and South America have been removed from residents in Asia, Europe, New Zealand and the USA [5,6,7,8,9,10,11,12,13]. In New Zealand, 50% of all tick importations that were intercepted at the border were associated with human travel [11]. There have also been cases of illness caused by tick-borne pathogens in recently returned travellers. More than 350 travel-associated cases of African tick bite fever (caused by *Rickettsia africae*) have been reported in Europe, USA, Australia, Argentina and Japan [14], and patients may not recall a tick bite, despite suffering from a tick-borne illness. For example, two USA residents developed skin lesions and flu-like symptoms within eight days of returning from Swaziland and were diagnosed with African tick bite fever, yet neither patient reported tick bites during the trips [15].

Whilst 20 tick species are considered endemic in the UK [16,17], UK residents can be exposed to non-native tick species whilst travelling abroad. As tick bites acquired overseas can present different health risks to those acquired in the UK, it is vital that existing public health guidance promotes the risks to both public health professionals and travellers. The following paper summarises imported ticks received by Public Health England’s passive Tick Surveillance Scheme (TSS) from humans with recent travel history and investigates the potential public health risk to UK residents bitten by ticks when travelling abroad.

## 2. Materials and Methods

Samples submitted to the TSS (see [18]) consisting of ticks likely acquired from outside of the UK between January 2006 and December 2018 were received from medical staff and members of the public. Upon arrival, specimens suspected to have been acquired overseas were frozen at −80 °C for 48 hours. Records were classed as imported if the recorder clearly stated that the tick had been acquired outside of the UK, the species was not endemic to the UK, or the tick could not have been acquired in the UK based on the level of engorgement and supplied travel information. All submissions of endemic species with a recent travel history that could not be confirmed as definitely acquired outside of the UK were classed as possible importation events, as local acquisition of the ticks could not be ruled out.

To identify species, keys for European ticks were initially used [19,20,21], with additional keys consulted for non-European species (e.g. [22]). Tick experts in the country of origin were contacted to verify specimens where necessary. The identification result was relayed to the person submitting the tick, as well as signposting to information about possible tick-borne diseases that are known to be transmitted by the tick species in the country of origin, and where necessary, follow-up questions were used to obtain more information about the tick encounter and the health of the person who had been bitten.

Following identification, a review of published literature was conducted using PubMed to further understand the ecology and potential public health risk following a bite from each tick species. First, searches were carried out for each tick species in the country of origin, for example ‘*Ixodes ricinus* AND France’, and then pathogens, for example ‘*Ixodes ricinus* AND France AND *Borrelia burgdorferi’*. The literature review of pathogens detected in ticks focused on articles that were published in English between January 2010 and March 2019, as well as the references cited therein. The relevance of articles identified by the database search (>900) were assessed first by their titles and abstracts, followed by an in-depth review of tick ecology and pathogen information.

## 3. Results

### 3.1. Tick Surveillance Scheme Imported Ticks

**Figure 1:**
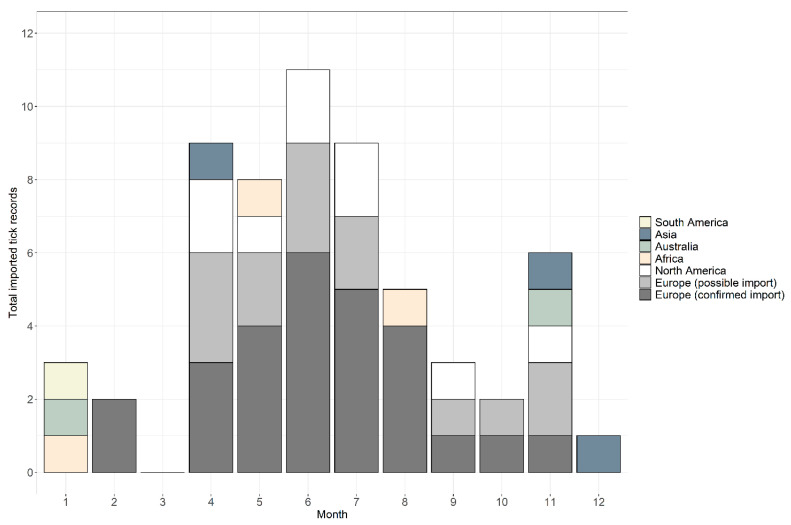
Seasonality of tick records received by the Tick Surveillance Scheme from people with a history of travel that were acquired in South America (yellow), Asia (blue-grey), Australia (green), Africa (beige) and North America (white), and possible imports from Europe (light grey) and confirmed imports from Europe (dark grey).

Between January 2006 and December 2018, 59 records were received from people with a recent travel history (Table 1). Records were comprised of 66 individual ticks belonging to 16 species, plus two damaged specimens that could only be identified at the genus level. In total, 76% of records (*n* = 45) were confirmed as imported and 24% (*n* = 14) were possible imports, where it was not possible to determine whether an endemic species received from someone with recent travel history was acquired abroad or in the UK. Considering only confirmed imported tick records, records were received in 2006 (*n* = 1), 2008 (*n* = 1), 2012 (*n* = 1), 2013 (*n* = 2), 2014 (*n* = 3), 2015 (*n* = 9), 2016 (*n* = 10) and 2017 (*n* = 10), and eight records in 2018. Possible imported records were received during 2013 (*n* = 2), 2015 (*n* = 2), 2016 (*n* = 2), 2017 (*n* = 3) and 2018 (*n* = 5). Across the years, ticks were removed from people with a history of travel during every month apart from March; the highest numbers were removed in June (*n* = 11), followed by July (*n* = 10), April (*n* = 9) and May (*n* = 8), totalling 63% of all records (see Figure 1 for a breakdown of confirmed and possible imported tick records). In addition, 20% of records (*n* = 12) were removed from travellers between November and February; half of these records originated from Southern Hemisphere countries (Figure 1).

**Figure 2:**
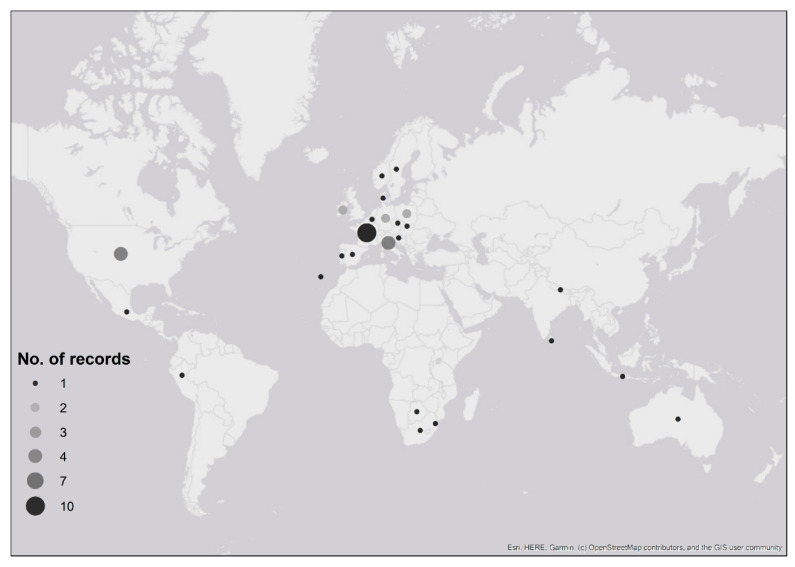
Map showing recent travel history of people submitting confirmed and possible imported tick records to Public Health England’s Tick Surveillance Scheme between January 2006 and December 2018. The numbers of records received from each country are indicated by the size of the circle. Two records are not shown: one record that was acquired in either Holland, Germany or France; another record that was acquired in either the Canadian Rockies or north-western USA (see Table 1). Maps have been reproduced with permission from Ordnance Survey on behalf of Her Majesty’s Stationery Office, © Crown Copyright and database right. 2020. All rights reserved. Ordnance Survey License number 100016969/100022432.

Ticks were received from people who had recently travelled to six continents: 70% of records were associated with travel in Europe, 15% from North America, 5% from both Africa and Asia, 3% from Australia and 2% from South America (Table 1; Figure 2). Confirmed imported ticks were removed from people that had recently travelled to 22 countries (Figure 2, Table 1), and possible imported ticks were removed from people with recent travel history to eight European countries (Figure 2, Table 1). There were two instances where the country of origin could not be determined as travel through multiple countries was reported (Table 1). One record, *Dermacentor marginatus,* was likely acquired either in Holland, Germany or France (although *D. marginatus* is not present in Holland (see [23])), whilst the second record, *Dermacentor andersoni*, came from either the Canadian Rockies or Washington State in north-western USA; despite the country of origin being unknown, both records were confirmed importation events as neither species is endemic to the UK. Exact locations where ticks were likely acquired were provided for 54% of records (*n* = 32), whilst regional/country information was provided for 46% (*n* = 27) of records.

In total, 16 tick species belonging to six genera were identified. Two species endemic to the UK were received: *Ixodes hexagonus* and *Ixodes ricinus*. In addition, fourteen non-native species were identified: *Amblyomma americanum*, *Amblyomma cajennense*, *D. andersoni*, *Dermacentor auratus*, *D. marginatus*, *Dermacentor variablis*, *Haemaphysalis hystricis*, *Hyalomma lusitanicum*, *Hyalomma truncatum*, *Ixodes holocyclus*, *Ixodes pacificus*, *Rhipicephalus appendiculatus*, *Rhipicephalus gertrudae* and *Rhipicephalus sanguineus* s.l. The most common tick species received was *I. ricinus*; there were 30 confirmed records and 12 possible records, representing 54% of all records, followed by *A. americanum*, which represented 8% (*n* = 5) of records. Nymphs were the most common (53% of all ticks) life stage received, followed by females (31%) and males (16%); no larvae were received. The ecology of each tick species, including distribution, habitat, hosts and seasonality, is presented in Table 2. Whilst human biting by the majority of the received species is commonly reported, human infestation of *Hy. lusitanicum*, *Hy. truncatum* and *H. hystricis* is considered rare or occasional (Table 2).

All records involving the importation of species endemic to the UK (*n* = 35) were acquired in Europe (Figure 2): 21 records (60%) were definitively confirmed as imported, whilst 14 records (40%) were possible imports (Table 1). There was one confirmed import of *I. hexagonus* from Portugal and a possible import of *I. hexagonus* from Madeira. Six confirmed *I. ricinus* imports originated from France, three from Germany, two each from Italy, Norway and Sweden and one each from Czechia, Ireland, Poland, Slovenia and Spain. In addition, four possible imported records were received from France, two each from Ireland, Italy and Poland and one from Germany.

### 3.2. Literature Review of Pathogens Detected in Ticks in the Native Country

Literature reviews for pathogens infecting tick species in the country of origin highlighted nine of the received species (*Ixodes ricinus*, *D. marginatus*, *R. sanguineus*, *Hy. lusitanicum*, *A. americanum*, *D. variabilis*, *D. andersoni*, *I. pacificus*, *I. holocyclus*), comprising 81% of all records, are known to transmit at least one pathogenic organism to humans; the most important pathogens in terms of human health in the country where the imported tick was likely acquired are shown in Table 2.

#### 3.2.1. Europe—*Ixodes Ricinus*

The most common pathogen detected in *I. ricinus* in Europe is the spirochete *Borrelia burgdorferi* sensu lato (s.l.), the causative agent of Lyme borreliosis (prevalence 0.06–46.6% [135]). Despite only being prevalent in the Northern Hemisphere, Lyme borreliosis is the most common tick-borne disease in the world [24]. It is transmitted by ticks belonging to the *I. ricinus* complex across Europe and North America, as well as in parts of northern Africa and Asia. The true incidence of infection in Europe is difficult to quantify, however, as Lyme borreliosis is not a notifiable disease. In Europe, the primary vectors of Lyme borreliosis are *I. ricinus* and *I. persulcatus*, but laboratory evidence suggests that *I. hexagonus* is also an efficient vector of *B. burgdorferi* s.l. [136,137].

There are multiple genospecies of *B. burgdorferi* s.l. circulating in Europe: *Borrelia afzelii, Borrelia bissettiae, Borrelia bavariensis* (formerly B. garinii OspA serotype 4), *Borrelia burgdorferi* sensu stricto (s.s.), *Borrelia carolensis*, *Borrelia finlandensis*, *Borrelia garinii*, *Borrelia lusitaniae*, *Borrelia spielmanii*, *Borrelia turdi* and *Borrelia valaisiana* [135,138]. Throughout Europe, the prevalence of genospecies varies geographically [138], which is likely driven by genospecies associated with different reservoir hosts. For example, whilst *B. afzelii* is associated with small mammals [139,140,141], ground-foraging birds are competent reservoirs of *B. garinii* and *B. valaisiana*, but are not able to maintain *B. afzelii* [142,143,144]. Not all genospecies are pathogenic, and in Europe at least five genospecies are well recognised as being pathogenic to humans—*B. afzelii*, *B. bavariensis*, *B. burgdorferi* s.s., *B. garinii* and *B. spielmanii* [145,146,147]. A meta-analysis investigating the prevalence of genospecies in questing *I. ricinus* across Europe found that *B. afzelii* (46.6%) and *B. garinii* (23.8%) were the most common genospecies, followed by *B. valaisiana* (11.4%), *B. burgdorferi* s.s. (10.2%), *B. lusitaniae* (7.0%), *B. bavariensis* (2.0%), *B. spielmanii* (1.7%), *B. finlandensis* (0.2%) and *B. bissettiae* (0.06%) [135]. Focusing on *I. ricinus* in the countries where recorders had a recent history of travel and subsequent tick bite, the most common genospecies reported in ticks, *B. afzelii*, *B. burgdorferi* s.s. and *B. garinii*, have been recorded from all focal European countries. In addition, other pathogenic species have been found: *B. bavariensis* in *I. ricinus* from Czechia and Germany, and *B. spielmanii* in Czechia, Denmark, France, Germany, Poland and Sweden.

Tick-borne encephalitis (TBE) is a severe neurological disease caused by a flavivirus transmitted by ticks in parts of Europe and Asia [148], although infection can be acquired to a lesser extent through consumption of raw milk from infected animals [149]. Tick-borne encephalitis virus (TBEV) is the most important tick-borne arboviral disease in Europe; it is endemic in 27 European countries and is expanding northwards and to higher altitude as a result of numerous factors including warming temperatures [150,151,152]. Adults were traditionally considered most at risk of TBE infection as the prognosis worsens with age, whereas children suffer milder symptoms [153,154,155,156,157]. A recent review of data from the European Centre for Disease Prevention and Control (ECDC), however, found that there is a greater risk of long-term cognitive impairment in children following TBE infection [158]. There are three subtypes of TBEV: European, Siberian and Far Eastern. The European subtype is transmitted by *I. ricinus*, whilst the other two are transmitted by *I. persulcatus* [155] (see Lindquist & Vapalahti, 2008). The virus is transmitted to a susceptible tick when it feeds adjacent to an infected tick, a process known as co-feeding [159], and key wildlife hosts are required for TBEV to persist in a population [160,161]. In southern Germany and Slovakia, most TBE cases occurred during June, July and August [162,163], which is likely linked to increased human activity during the summer period, despite reduced tick numbers [164]. Between 2012 and 2016, 12,500 cases of TBE were reported from 23 European countries, and of these, Czechia and Lithuania accounted for 38.6% of all reported cases [165]. Prevalence < 0.1–5% [166]. In the focal countries for the current study, TBEV has been found in *I. ricinus* in all countries apart from Ireland and Spain (Table 2), and the highest number of cases of TBEV have been reported from Czechia [165]. To date, five imported cases of TBE have been reported in the UK, although the country where the infection was acquired is unknown [165].

#### 3.2.2. Europe: *Dermacentor marginatus* in France, Germany and Italy

Tick-borne lymphadenopathy (TIBOLA) or *Dermacentor*-borne necrosis erythema lymphadenopathy (DEBONEL) is caused by three species of *Rickettsia*: *R. slovaca*, *R. raoultii* and *R. rioja* [167,168,169,170,171,172]. Cases of infection have been reported from Bulgaria, France, Hungary, Italy, Portugal and Spain [168,173,174,175,176,177,178,179]. Compared to Lyme borreliosis, TIBOLA/DEBONEL is a relatively rare and mild infection, but awareness of symptoms is still important [180]. In Europe, *D. marginatus* is considered to be the main vector of TIBOLA/DEBONEL, and the peak in diagnosed cases coincides with the peak in adult activity [175]. The prevalence of *R. slovaca* infecting *D. marginatus* is between 15.7% and 50% [33,173,181,182,183,184,185], whilst the prevalence of *R. raoultii* is lower at 8.3–8.4% [33,184]. Of the focal countries in the current study, infection with *R. slovaca* has been reported in *D. marginatus* from France, Germany and Italy, and *R. raoultii* has been detected in *D. marginatus* from Italy.

#### 3.2.3. Europe: *Rhipicephalus sanguineus* s.l. in Croatia and Italy

The most frequent rickettsiosis in Europe is Mediterranean spotted fever (MSF, also named fièvre boutonneuse), caused by *Rickettsia conorii*, and cases have been reported from much of Western and Southern Europe, as well as parts of Eastern Europe [175]. Mortality can occur, and a fatality rate of 32.3% of hospitalized patients with severe morbidity in southern Portugal has been reported [186]. As *Rh. sanguineus* is thought to act as both the vector and reservoir of the rickettsia, some mammals present in the Mediterranean may also be reservoir hosts [175]. Although *Rh. sanguineus* has a low probability of biting humans, most cases of infection are diagnosed in the spring and summer when *Rh. sanguineus* activity peaks [175]. In an MSF-endemic region in Spain, however, only one out of 4049 ticks was infected [187]. Very low infection prevalence (<1%) has been reported [188,189], but can be higher (see [190]) As other *Rickettsia* species were more prevalent, the authors suggested that many of the cases that had been attributed to MSF may have actually been caused by other *Rickettsia* species [187]. For example, *Rickettsia conorii israelensis,* which has also been detected in European *Rh. sanguineus* populations, results in a sudden fever, accompanied by headache, rash, and gastrointestinal symptoms [191]. Mortality has been reported in less than a third of patients [191], but is higher than that reported for MSF, suggesting *R. conorii israelensis* is more virulent than *R. conorii* [192]. Another Spotted Fever group rickettsia which has been detected in multiple species belonging to the *Rhipicephalus* genus [193], including *Rh. sanguineus,* is *R. massiliae*. Symptoms include fever, headache, muscular pain, rash and eschar [190,194,195,196], and in Italy and France, *R. massiliae* is considered the causative agent of a MSF-like illness in patients [190,194,195,196]. During the current study, two imported *Rh. sanguineus* were received; one from Italy and one from Croatia. Whilst to date, there is no information on pathogen prevalence in *Rh. sanguineus* in Croatia, *R. conorii, R. conorii israelensis* and *R. massiliae* have all been detected in *Rh. sanguineus* from Italy.

#### 3.2.4. Europe: *Hyalomma lusitanicum* in Spain

Crimean Congo haemorrhagic fever virus (CCHFV) is caused by a member of the Nairovirus genus and is transmitted via bites from *Hyalomma* species (particularly *Hy. marginatum marginatum* but also *Hy. lusitanicum*), or following contact with infected body fluids or tissues [197]. To date, two studies have detected CCHFV in *Hy. lusitanicum* at a prevalence of 3.2% [45,198] feeding from red deer (*Cervus elaphus*) in Spain between 2010 and 2015, suggesting that CCHFV is circulating in southwestern Europe [45,198], but whether *Hy. lusitanicum* is a vector of the virus remains unclear. During 2016, two autochthonous cases of CCHFV were reported in Spain [199], followed by a subsequent fatal case reported in August 2018 [200]. Until the end of August 2020, there have been three autonomous cases of CCHF in Spain and Bulgaria, including a fatality [201,202,203]. To date, there have been three CCHFV cases reported in the UK. First, in 1997, a suspected case of CCHFV was diagnosed in a UK resident recently returned from Zimbabwe [204]. In 2012, there was a fatal CCHFV case in a UK national returning from Afghanistan [205]. Finally, in June 2014, there was a case in a UK national that had received a tick bite whilst in Bulgaria [206].

#### 3.2.5. North America: *Amblyomma americanum* in USA

In the USA, Lyme borreliosis is caused by the genospecies *B. burgdorferi s.s.,* and in the north-eastern USA where Lyme borreliosis is most prevalent, *I. scapularis* is the primary vector, whilst in the western states, the bacteria are vectored by *I. pacificus*. Infection of *A. americanum* with *B. burgdorferi* s.s. has been reported from a number of states [207,208,209,210], yet it is not considered a competent vector [211,212,213]. Furthermore, Lyme borreliosis-like symptoms have been associated with bites from *A. americanum* in some southern states when no trace of antibodies to *B. burgdorferi* s.l. have been detected in patients [214,215]. The spirochete responsible has been identified and termed ‘*Borrelia lonestari*’ [216], and has been detected at low prevalence (0.35–9.1% [216,217,218,219,220,221,222,223,224,225,226,227]) in questing *A. americanum* from multiple locations [216,217,218,219,220,221,222,223,224,225,226,227]). The resulting illness is named Master’s disease or southern-tick-associated rash-illness (STARI), and is clinically indistinguishable from the early stages of Lyme borreliosis [228]; however, the public health significance of *B. lonestari* remains unclear.

*Ehrlichia chaffeensis* and *E. ewingii* are gram-negative bacteria that are the causative agents of human ehrlichiosis [229,230]. Whilst ehrlichiosis caused by *E. chaffeensis* is considered to be underreported and probably as prevalent as Rocky Mountain spotted fever (see below), less than 20 cases of *E. ewingii* have been documented, and are mostly reported in immunosuppressed patients [52,231]. The prevalence of *E. chaffeensis* in ticks is higher (0.6–19%) [217,220,221,222,232,233,234,235,236,237,238,239] than *E. ewingii* (0.24–7.1%) [217,220,221,222,233,234,235,236,238]. It is thought that *E. chaffeensis* and *E. ewingii* are maintained in cycles involving a wide variety of competent vertebrate reservoir hosts, such as white-tailed deer, and *A. americanum* as the primary vector [52,235,240]. Laboratory experiments have demonstrated transmission of *E. ewingii* from *A. americanum* to dogs, highlighting the importance of *A. americanum* in maintaining and transmitting ehrlichiae to hosts [241]. In 2006, Panola Mountain Ehrlichia (PME), caused by a disease agent similar to *Ehrlichia ruminantium*, was discovered in a goat from Panola Mountain State Park in Georgia [242] and was later associated with a report of human illness following a bite from an *Amblyomma* nymph also acquired at the State Park [243]. Furthermore, *A. americanum* infected with PME have been collected from ten states on the east coast of the United States between 1998 and 2006, suggesting the infection has an extensive distribution throughout the *A. americanum* range and has not been recently introduced [244].

#### 3.2.6. North America: *Dermacentor variabilis* in USA

*Dermacentor variabilis* is a primary vector USA [245,246]. Caused by the bacteria *Rickettsia rickettsii*, RMSF is the most common tick-borne rickettsial disease in the USA [247]. Severe illness can develop if symptoms remain untreated, and fatalities are possible [247]. Low prevalence rates of less than 2% in *D. variabilis* populations [248,249] have led some authors to question whether other tick species may play a more important vectoral role than *D. variabilis* [249]. For example, *Rh. sanguineus* was the cause of an outbreak of RMSF in eastern Arizona [250]. Furthermore, whilst *A. americanum* historically has not been considered to be a major vector of RMSF [251], much of the distribution overlaps with *D. variabilis* [252], and as both species feed on similar hosts, there is potential for *A. americanum* larvae to become infected with *R. rickettsii* either whilst feeding on an infected host or cofeeding next to an infected *D. variabilis* [252]. Under laboratory conditions, *A. americanum* has acquired the infection from guinea pigs, maintained infection throughout moulting and transmitted the bacteria to susceptible hosts during subsequent feeding, suggesting that under laboratory conditions, *A. americanum* is capable of acquiring, maintaining and transmitting *R. rickettsii* [252]. To date, however, there is no evidence to suggest that *A. americanum* is a competent vector of *R. rickettsii* in the USA. *Rickettsia montanensis* is another spotted fever group Rickettsia that has been detected in *D. variabilis*. Studies in the USA and Canada have found a mean infection prevalence of *D. variabilis* with *R. montanensis* of 1.2–10.5% [56,59,248,253,254,255,256,257,258,259]. In addition, a child from Georgia, USA developed an afebrile rash illness after being bitten by an individual *D. variabilis* infected with *R. montanensis*, suggesting the possibility that *R. montanensis* may be a human pathogen [260].

#### 3.2.7. North America: *Ixodes pacificus* in USA

*Ixodes pacificus* is the primary vector of Lyme disease in western USA [110], and the highest regional incidence occurs in north-western California, where dense oak woodlands support high densities of *I. pacificus* [261]. In California, cases of Lyme borreliosis peak between May and July, following the nymph peak between April and June, suggesting nymphs are responsible for most cases in California [72].

*Borrelia miyamotoi* belongs to the relapsing fever group of *Borrelia*, and is distinctly related to *B. burgdorferi* s.l. It is transmitted by the same tick species as *B. burgdorferi* s.l., namely *I. ricinus* and *I. persulcatus* in Europe, and *I. scapularis* and *I. pacificus* in the USA. Three distinct genotypes of *B. miyamotoi* have been identified in the USA, Europe and Japan [262,263,264], and all three groups include strains that are pathogenic to humans [265]. Unlike the spirochetes that cause Lyme borreliosis, *B. miyamotoi* spirochetes are able to be maintained in ticks via both transstadial and transovarial transmission [266,267]. The first human cases of infection with *B. miyamotoi* were reported in Russia in 2011 [268], and further cases have been reported from elsewhere in Europe (Netherlands and France) and the USA [269,270,271,272]. A study of people bitten by ticks which tested positive for *B. miyamotoi* who went on to develop clinical disease estimated transmission efficiency in humans to be 8.3% (2/24 patients), compared with 4.4% (3/68) of humans bitten by ticks infected with *B. burgdorferi* s.l. who went on to develop symptoms [273,274]. In the USA, white-footed mice (*P. leucopus*) are important hosts of the bacteria [266], and a high prevalence of infection (58%) has been detected in wild turkeys (*Maleagris gallopavo*) [275]. Prevalence rates of infection in ticks are significantly lower (0.4–5% [276,277,278,279,280,281,282]) than *B. burgdorferi* s.l. (0.2–24.7% [64,261,276,277,279,281,283,284,285,286,287,288,289,290,291,292,293,294,295]), although a comparable prevalence of *B. miyamotoi* and *B. burgdorferi* in adult *I. pacificus* in California suggests that there is a similar risk of exposure to both species [276]. As well as *I. pacificus* from USA, *B. miyamotoi* infection has been reported in *I. ricinus* from Czechia, France, Germany, Norway, Poland, Spain and Sweden (Table 2). In Europe, bank voles (*Myodes glareolus*) and yellow-necked mice (*Apodemus flavicollis*) are reservoirs for *B. miyamotoi* [263,296,297], and whilst bacteria have been detected in engorged *I. ricinus* feeding on wild boar (*Sus scrofa*), roe deer (*Capreolus capreolus*) and a blackbird (*Turdus merula*), it is unknown whether these species are reservoir hosts of *B. miyamotoi* [298].

*Anaplasma phagocytophilum* is an obligate intracellular bacterium that is responsible for causing human granulocytic anaplasmosis (HGA), a moderate flu-like illness in humans. In the USA, HGA is the second most important tick-borne disease after Lyme borreliosis, and is a notifiable infection, although many infections may result in minimal or no clinical manifestations [299]. Small mammals, which feed immature tick stages, are the primary reservoirs for *A. phagocytophilum* [300,301,302]. In eastern USA, the main vector of HGA is *I. scapularis*, whilst *I. pacificus* is considered the main vector in western parts of the USA [303,304]. Further, the prevalence of *A. phagocytophilum* in *I. pacificus* is significantly lower (0–11%) than the prevalence in *I. scapularis* (0-51%) [277,283,285,288,295,300,305,306,307].

#### 3.2.8. North America: *Dermacentor andersoni* in USA/Canada

Along with being a primary vector of RMSF [308], *D. andersoni* is also the principal vector of Colorado tick fever virus, which is found in western USA. The disease is considered under-reported, as non-specific symptoms may be misdiagnosed as other infections including RMSF [309]. Whilst producing a febrile illness in humans, the virus does not cause illness in its natural reservoir hosts. It was first detected in Montana, where adult *D. andersoni* were found to be infected with the virus, and infection was also present in ground squirrels, the preferred host for immature stages of *D. andersoni* [310,311]. A prevalence of 6.6% to 21% in *D. andersoni* has been reported [312,313]. Further, clinical cases of Colorado tick fever peak between May and July, concurrently with the peak of ticks [314].

*Dermacentor andersoni* has been associated with tick paralysis, with rapidly ascending paralysis occurring five to seven days after tick attachment. The tick often attaches to the head, and so can be hidden by hair; removal of the tick leads to an immediate improvement in symptoms [315]. In a review of 33 cases of tick paralysis that were reported to the Washington State Department of Health between 1946 and 1996, *D. andersoni* was identified as the culpable species [316].

#### 3.2.9. North America: *Amblyomma cajennense* Sensu Lato in Mexico

There are no studies published to date reporting the association of *A. cajennense* with human disease in Mexico. In South America, however, *A. cajennense* is considered to be the most important vector of *R. rickettsii* (the causative bacteria of RMSF, also called Brazilian spotted fever (BSF) in Brazil [99]). Considered to be the deadliest of the spotted fevers in the world, BSF has a fatality rate of 31.5%, compared with 5–10% fatality for RMSF in the USA [317,318]. A laboratory experiment found that it was possible for transovarial transmission from a female *A. cajennense* to her offspring, but only some of the eggs became infected, suggesting that *R. rickettsii* is not able to be maintained by transovarial transmission only [319]. In addition, *A. cajennense* larvae that fed on guinea pigs experimentally infected with *R. rickettsii* were less susceptible to infection with *R. rickettsii* than other tick species [320].

#### 3.2.10. Australia: *Ixodes holocyclus*

*Ixodes holocyclus* is responsible for the majority of (though not all) cases of tick paralysis in Australia [316]. Tick paralysis is a rare but potentially fatal condition. Between 1904 and 1945, there were 20 fatalities reported in Australia due to tick paralysis, with 70% of all deaths being children, but since 1945, there have been no further fatalities [321]. Ticks do not secrete detectable levels of the paralysis-inducing toxin until three days of feeding on a host [73]. As such, clinical signs of tick paralysis do not become apparent until three days following a tick bite [322,323]. Prompt tick removal is an important part of patient treatment [324], although, following removal of *I. holocyclus*, there can be a deterioration in the health of the patient [321]. Whilst nymphs may cause a mild paralysis, females are responsible for the most severe symptoms, and a bite from a single infected female tick is sufficient to cause paralysis [74,322,325]. Cases of paralysis occur year-round, but are most common in spring and summer [323]. It appears that children up to 5 years old are most affected [74]. Furthermore, *I. holocyclus* is usually found on the scalp and paralysis is less likely to occur if ticks are attached to other parts of the body [74].

#### 3.2.11. Africa: *Hyalomma truncatum* in Botswana

Whilst there have been no published studies looking at the pathogen prevalence in *H. truncatum* collected in Botswana to date, horizontal transfer of CCHFV from larval *H. truncatum* to laboratory mice has been reported [326], and transovarial and sexual transmission of CCHFV in *H. truncatum* has been seen in laboratory studies [327,328]. In addition, whilst Rift Valley fever virus is predominantly associated with mosquito transmission or contact with infected blood/body fluids, it has been suggested that *H. truncatum* is a possible vector of the virus [329,330]. Horizontal and transstadial transmission has been demonstrated [329], and the geographical distribution of *H. truncatum* correlates with the incidence of Rift Valley fever virus [330]. *Rickettsia aeschlimannii* and *R. africae* have been detected in *H. truncatum* [331,332,333,334,335], although there does not appear to be confirmed evidence of transmission from laboratory experiments. The prevalence of *R. aeschlimannii* in *H. truncatum* was lower compared to other *Hyalomma* species, suggesting that *H. truncatum* is not the principal vector of the bacteria [331].

#### 3.2.12. Africa: *Rhipicephalus gertrudae* in South Africa

To date, there is no information on whether *Rh. gertrudae* is involved in the transmission of any pathogens to humans in South Africa or further afield.

#### 3.2.13. Africa: *Rhipicephalus appendiculatus* in Eswatini

Although *Rh. appendiculatus* is a known vector of diseases affecting cattle in Southern Africa, for example, East Coast fever [336], there have been no published studies to date to suggest it to vector any diseases which can be transmitted to humans in Eswatini or further afield.

#### 3.2.14. Asia: *Haemaphysalis hystricis* in Java, Indonesia

Whilst to date there have been no studies published looking at the pathogen prevalence in *H. hystricis* collected specifically in Java or across Indonesia, the findings of studies that have been conducted in other Asian countries suggest that *H. hystricis* could act as a vector in Java. *Haemaphysalis hystricis* is considered to be one of the most important vectors of *Rickettsia japonica*, the bacteria responsible for Japanese spotted fever [120], and infected ticks have been found in Japan and China [337,338] (reviewed in Mahara, 1997; Lu *et al.*, 2017). Following the diagnosis of a patient with Japanese spotted fever in Fukuoka Prefecture in Japan, an investigation of ticks found in the area where it was thought that the patient most likely acquired the tick bite found larvae were infected with *R. japonica* [339]. Similarly, in the Hubei province of China, *R. japonica* was detected in a single *H. hystricis* [338]. Focusing on other infections, *Coxiella* species have been detected in *H. hystricis* in Thailand, although infection prevalence in *H. hystricis* is thought to be lower than in other *Haemaphysalis* species also present in Thailand [340]. Following on from this, a study of *H. hystricis* from Malaysia suggested the possibility of infection with *C. burnetii* based on the phylogenetic clustering of the bacteria [341]. Finally, whilst *Borrelia burgdorferi* s.l. has not been detected in *H. hystricis* from China [115], *Borrelia* sp. closely related to the relapsing fever group (e.g. *Borrelia miyamotoi*) have been detected in *H. hystricis* from Malaysia [116], although it should be noted that the bacteria was only detected in a single tick.

#### 3.2.15. Asia: *Dermacentor auratus* in Nepal and Sri Lanka

To date, there has been no investigation into pathogens found in *D. auratus* in either Nepal or Sri Lanka. Kyasanur forest disease and Lanjan virus have been isolated in questing *D. auratus* in India and Malaya, respectively [131,342], although it is unclear whether *D. auratus* also transmits the pathogens. A study of ticks removed from wild boar in Thailand found *D. auratus* to be infected with *Rickettsia raoultii* (3/11 = 27%) and *Francisella*-like endosymbionts (2/11 = 18%), whilst *Coxiella* bacteria were also investigated but not detected [132].

## 4. Discussion

Between January 2006 and December 2018, Public Health England’s Tick Surveillance Scheme (TSS) received 59 records from humans with recent travel history, comprised of 66 individual ticks belonging to 16 tick species that were associated with travel outside of the UK. Records were submitted by people with recent travel history to 25 different countries, with 70% of confirmed and possible imported records being associated with travel to Europe. Although less than five percent of total records received by the TSS are imported ticks [18], the findings suggest that UK travellers are exposed to a variety of tick species when abroad. Whilst it was not possible to test the ticks for pathogens in the current study, literature reviews suggest that just over half (9/16) of the received tick species are known to vector at least one tick-borne pathogen in the country of acquisition, suggesting that travellers exposed to ticks may be at risk of being bitten by a species that is a known vector, which could have implications for the transmission of novel tick-borne diseases to UK travellers.

Whilst there are several records of non-native ticks entering the UK previously on animal hosts, including dogs [44,343,344,345,346,347,348,349,350,351,352], horses [353], migratory birds [354,355] and reptiles [356,357,358], the current study is an extensive report of tick exposure to UK travellers submitted to the TSS. Reports of two records included in the current study have been published previously [6,359]. First, a female *I. holocyclus* was detected on a traveller recently returned from Australia who presented with swelling at the bite site and was prescribed antibiotics as an infected mole or wart was suspected [6]. In the second case, a female *D. marginatus* was received from a patient with no history of travel experiencing swelling, swollen glands and flu-like symptoms [359]. As *D. marginatus* is not endemic in the UK, further investigations revealed that the patient had had contact with several European visitors in the month preceding the bite, and a family member had driven her car through Holland, Germany and France the week before, so it was suggested that the tick was imported by one of the travellers [359]. Such an event highlights the importance for clinicians to obtain substantial information from patients presenting with tick bites. Investigations should include questions regarding extended travel history, including several weeks prior to tick removal, as several non-native tick records were received where the date of removal was several weeks after the person had returned from abroad. In addition, questions on contact with visitors who have recently been overseas should also be considered.

Some of the imported species received by the TSS have been reported as imported in the UK previously. Two days after returning from a two-month trip to Missouri, an 84-year-old male presented to his general practitioner in Northern Ireland, and an almost fully fed female *A. americanum* was detected [8]. Another study described 16 tick species removed from UK domestic and international travellers; six non-native species detected in the paper are reported in the current study (*A. americanum, A. cajennense, D. variabilis, D. marginatus, I. scapularis* and *Rh. sanguineus*), whilst six additional non-native species were also described (*A. maculatum, A. hebraeum, A. variegatum, H. concinna, Hy. marginatum marginatum* and *Hy. marginatum rufipes*), as well as four species acquired within the UK (*D. reticulatus, H. punctata, I. ricinus* and *I. hexagonus*) [13]. Whilst *I. ricinus* was the most common species detected, 25 of 28 records were acquired within the UK, whereas of tick records acquired from outside of the UK, the most common species detected was *D. marginatus* (four records), acquired in France, Italy, Greece and Romania [13]. Other published studies have described non-native tick species detected on imported dogs in the UK, including *Hy. lusitanicum* [346], *D. variabilis* [351], *I. holocyclus* [343] and *Rh. sanguineus* [351]. Four of the species reported in the current study, however, have been detected on UK travellers for the first time: *H. hystricis*, *Hy. truncatum*, *Rh. appendiculatus* and *Rh. gertrudae.* As imported tick species can transmit a range of pathogens, UK travellers may be exposed to novel pathogens which are unfamiliar to public health professionals in the UK. As such, issuing appropriate public health guidance on tick awareness and avoidance prior to travel could reduce the risk of acquiring tick bites and subsequent tick-borne infections whilst overseas.

Confirmed and possible imported ticks were removed from people with recent travel history in all months apart from March. Sixty-three percent of records detailed ticks removed between April and July, which may reflect travelling activity, as most travel abroad by UK residents occurs during these months [360]. Seasonality of adult and nymph *I. ricinus* in the UK and Europe is highest between April and June [361,362,363], however, so it is also possible that the peak in imported ticks between April and July may be a consequence of 54% of all records being *I. ricinus*. It should be noted, however, that 20% of imported records were received between November and February, when *I. ricinus* activity is at its lowest in the UK [18]. The results from the current study suggest that the risk of being bitten by a tick whilst travelling occurs throughout the year, but is likely to vary depending on travel destination, so advice on tick awareness, removal and tick-borne infections should be provided to travellers year-round, with special attention paid during late spring and early summer when tick bite risk is highest in the most common destinations for UK travellers.

Seventy percent of ticks received by the TSS from people with a recent travel history were associated with recent travel to Europe, and as Europe is a common destination for UK travellers, the risk of ticks and tick-borne diseases being imported from Europe is higher. The predominant species received from people recently returned from Europe was *I. ricinus*, which is also the most common tick species found throughout the UK [18]. In Europe, *I. ricinus* is the predominant vector of *B. burgdorferi* s.l., the causative agent of Lyme borreliosis, which is the most common tick-borne infection in Europe with approximately 65,000 cases reported annually between 1987–2006 [364,365,366]. Overall, the highest incidence of Lyme borreliosis has been reported in eastern and central Europe, and decreases from east to west, although local incidence can vary [135,367,368,369,370,371]. Lyme borreliosis should be considered in patients returning from Europe with a history of tick bites, yet whether there is an increased or reduced risk of UK travellers being bitten by *B. burgdorferi*-infected *I. ricinus* in European countries compared with the UK will be dependent upon the country of travel. As the incidence of Lyme borreliosis is higher in eastern and central Europe [135,367,368,369,370,371], travellers to these regions in particular should be made aware of the increased risk and symptoms of Lyme borreliosis.

Along with Lyme borreliosis, TBE should also be considered in patients presenting with symptoms and a history of tick bites following travel to Europe. It is the second most important tick-borne disease transmitted by *I. ricinus* in Europe, and to date, infected *I. ricinus* have been detected from nine of the eleven European countries where recorders had a recent history of travel and subsequent tick bite. Several cases of travellers contracting TBE whilst abroad have been previously reported. Five imported cases of TBE were reported in the USA between 2000 and 2009 from patients who had travelled to Czechia, Sweden, Russia and China, and four out of five of the patients described having multiple tick bites whilst abroad [372]. Between 2012 and 2016, five imported cases of TBE were described in the UK, although the country of origin was unknown [165]. In 2011, two Dutch travellers returning from Austria were diagnosed with TBE; and similarly to the previous study, both patients reported tick bites [373]. In parts of Austria, TBEV is endemic, and the risk of a tourist acquiring the infection after spending four weeks in an endemic region is estimated as 1 in 10,000 and following this, it is estimated that 60 travel-associated (i.e. exported) cases of TBE were likely to occur over the whole summer period [374]. As the popularity of outdoor activities such as hiking and biking increases, people may be more exposed to tick bites than in the past [375]. The risk of acquiring TBEV infection when abroad, therefore, should be made clear to travellers from non-endemic regions [375]. Furthermore, the incidence of TBE is expanding northwards to higher latitudes and altitudes [150,151,152], and could result in increased human exposure to infected ticks in the future.

Whilst Lyme borreliosis is the most important tick-borne infection in Europe and North America, it is important to remember this is not the case in other countries, so patients with travel history to other continents may present with different symptoms. It is also important to consider that the same organism may have a different pathogenicity in a different country. An example of this is the causative agent of human granulocytic anaplasmosis (HGA), *Anaplasma phagocytophilum*. In the USA, HGA is a notifiable infection, and the second most important tick-borne disease (after Lyme borreliosis), with increasing infection incidence from 1.4 cases per million persons in 2000 to 17.9 cases per million persons in 2017 [376]. In comparison, only 70 cases have ever been reported in Europe and are sporadic [377,378]. It is unclear whether the small number of confirmed cases in Europe is due to under-reporting, under-diagnosis or low pathogenicity of *A. phagocytophilum* strains circulating in Europe [299].

## 5. Conclusions

Since 2006, 16 tick species removed from humans with a recent travel history were submitted to the TSS. Furthermore, literature reviews suggested that nine of the received species are known to vector at least one organism that is pathogenic to humans in the country of travel. Such findings suggest that travellers exposed to ticks may be at risk of being bitten by species known to transmit at least one pathogen. As 70% of received ticks were confirmed or suspected to have been acquired in Europe, and European countries are common destinations for UK travellers, the risk of imported ticks and tick-borne diseases from Europe is higher, and UK travellers should be made aware of the risk preceding and following European travel.

## Figures and Tables

**Table 1:** Ticks submitted to Public Health England’s Tick Surveillance Scheme from people with recent travel history outside of the UK between 2006 and 2018. The tick species, history of travel, number of records associated with each tick species (including confirmed (C) and possible (P) imported ticks) and number of each life stage identified are shown (no larvae were submitted).

Tick Species	Continent	History of Travel	No. Records	No. Confirmed/Possible Imported Records	Males	Females	Nymphs	Total Ticks
*Amblyomma americanum*	North America	USA	5	5/0	-	3	3	6
*Amblyomma cajennense* s.l.	North America	Mexico	1	1/0	-	1	-	1
*Amblyomma* species *	South America	Peru	1	1/0	-	-	1	1
*Dermacentor andersoni*	North America	USA/Canada	1	1/0	1	-	-	1
*Dermacentor auratus*	Asia	Nepal	1	1/0	-	-	1	1
		Sri Lanka	1	1/0	-	-	1	1
*Dermacentor marginatus*	Europe	Holland/Germany/France	1	1/0	-	1	-	1
		Italy	2	2/0	2	-	-	2
*Dermacentor variablis*	North America	USA	1	1/0	1	-	-	1
*Haemaphysalis hystricis*	Asia	Java, Indonesia	1	1/0	-	1	-	1
*Hyalomma lusitanicum*	Europe	Spain	1	1/0	1	-	-	1
*Hyalomma truncatum*	Africa	Botswana	1	1/0	1	-	-	1
*Ixodes hexagonus*	Europe	Madeira	1	0/1	-	1	-	1
		Portugal	1	1/0	-	1	-	1
*Ixodes holocyclus*	Australia	Australia	2	2/0	-	1	1	2
*Ixodes pacificus*	North America	USA	1	1/0	-	-	1	1
*Ixodes ricinus*	Europe	Czechia	1	1/0	-	-	1	1
		Denmark	1	0/1	-	1	-	1
		France	10	6/4	-	3	10	13
		Germany	4	3/1	-	1	4	5
		Ireland	3	1/2	-	-	4	4
		Italy	4	2/2	-	1	3	4
		Norway	2	2/0	3	1	1	5
		Poland	3	1/2	-	2	1	3
		Slovenia	1	1/0	-	-	1	1
		Spain	1	1/0	-	1	-	1
		Sweden	2	2/0	-	1	1	2
*Ixodes* species *	Europe	Belgium	1	0/1	-	1	-	1
*Rhipicephalus appendiculatus*	Africa	Eswatini	1	1/0	-	-	1	1
*Rhipicephalus gertrudae*	Africa	South Africa	1	1/0	1	-	-	1
*Rhipicephalus sanguineus* s.l.	Europe	Croatia	1	1/0	1	-	-	1
		Italy	1	1/0	-	-	1	1
**Total**			**59**	**45/14**	**11**	**21**	**36**	**68**

* indicates that the tick was damaged and could only be identified at the species level. Total numbers of records and ticks are shown in bold.

**Table 2:** Review of distribution, habitat and climate requirements, hosts, seasonality and commonality of human biting of each confirmed and possible imported tick species, along with the prevalence of the most common human pathogens detected in the focal tick species in the country where the tick was confirmed or suspected to have been acquired. References are mostly focused on countries listed in the history of travel, but literature searches were extended if there was no information available for the tick species in the country.

Tick Species	Distribution	Habitat and Climate Requirements	Hosts	Seasonality in Countries of Interest	Commonality of Human Biting
*Ixodes ricinus* (sheep or castor bean tick)	Most widespread tick across Europe, also present in parts of North Africa and Asia [24]	Deciduous and mixed forests, scrub, parks	Larvae: small mammals Nymphs: woodland and game birds Adults: large mammals, particularly deer [25,26,27,28,29,30,31]	Active year-round; peaks in larvae during June. Nymphs and females peak in May-June, with smaller peak in September [32]	Most common tick species found biting humans in Europe
*Dermacentor marginatus* (ornate sheep tick)	Found south of the Alps, stretching from Portugal across Southern Europe, into northern Africa and mountain region of central Asia [23,33]	Dry habitats with sparse vegetation, e.g., alpine and forest steppes and semi-desert regions; tolerant to a wide range of environmental conditions [34,35,36]	Larvae: small mammals Nymphs: small mammals Adults: larger mammals including horses, cattle, dogs, deer, sheep [34]	Larvae: June and July Nymphs: July and August Adults: March–May, recommences in September & October [37,38]	Adults are known to bite humans [23,39]
*Rhipicephalus sanguineus* s.l. (brown dog tick)	Most widely distributed tick species in the world; more commonly associated with Mediterranean areas in Europe [40]	Capable of surviving in temperate regions. Can survive inside buildings, particularly those housing dogs but also residential homes	Dogs are the primary host of all stages, although immature stages can feed on rodents and small mammals	Active in temperate regions between spring and autumn [40]. Can be active year-round inside properties	Low probability of biting humans, but can be a problem in infested residential homes [39,41,42,43,44]
*Hyalomma lusitanicum*	Limited to a narrow strip from Sicily to Portugal and North Africa [45,46]	Adapted to meso-Mediterranean climate summer conditions [47]	Larvae and nymphs: Mediterranean rabbits (*Oryctolagus cuniculus huxleyi*) Adults: ungulates, insectivores, carnivores [45,48,49]	Adult questing begins in March, peaks May–July then declines, with a small increase in September–October [47,50,51]	Human biting considered rare and accidental [49]
*Amblyomma americanum* (lone star tick)	Found in 39 states plus the District of Columbia in USA, stretches across eastern, south-eastern, mid-western and north-eastern USA [52,53]	Wooded areas with thick underbrush, as well as scrub and meadows, where the primary host (white-tailed deer, *Odocoileus virginianus*) resides	Larvae and nymphs feed on small- and medium-sized hosts, adults feed on medium- and large-sized mammals. Host range is vast, but white-tailed deer are the most important host and can feed all life stages—recent expansions in lone star tick populations due to increase in white-tailed deer populations [52]	Larval activity is highest during the summer, nymph and adult activity peaks April–June and then declines [52]	One of the most aggressive human-biting tick species, often the most abundant human biting species in many studies [52,53,54,55,56]
*Dermacentor variabilis* (American dog tick or wood tick)	Found in USA from Gulf of Mexico to New England and through the mid-western states to the east of the Rocky Mountains, with an independent population also found in California. Also present in parts of Mexico	Found in areas with limited tree cover, like brushy field habitat and scrubland [57]	Larvae and nymphs: small mammals Adults: dogs, cervids, ruminants	Two peaks in adult questing: mid-April to late May; July [58]	Adults are the only stage known to bite humans [59]
*Dermacentor andersoni* (Rocky Mountain wood tick)	Found throughout the western United States and is established in at least 14 states [60]	Found in semiarid and mountainous areas with woodland, scrub and grassy areas [60,61,62]	Larvae and nymphs: small mammals, e.g., rats, squirrels, chipmunks Adults: larger hosts such as cattle, deer, horses and elk [60].	Larvae and nymphs occur between March and October: larval peaks occur from June to July whilst nymphs peak from May to June [60]. Adults are active between February and November, with greatest abundance recorded from March to April [60]	Larvae and nymphs rarely bite humans
*Ixodes pacificus* (western blacklegged tick)	Found in western parts of the United States, particularly Washington, Oregon, California, Arizona, Nevada and Utah [63]	More nymphs found in warmer and drier hardwood-dominated woodlands compared with cooler and more humid woodlands dominated by redwoods [64]. Survival is limited by summer drought conditions; larvae that hatch in the early summer remain in behavioral diapause until the following spring to avoid desiccation [64,65]	Larvae and nymphs: birds (migratory and non-migratory), rodents and lizards Adults: birds and lizards, as well as larger mammals such as cats, dogs and ruminants [66,67,68]	Larval and nymphal infestation of birds and lizards peaks in April and May [66,69,70]. Questing nymphs active March–September, peaking in May and June, whilst adults are active October–June with peaks in November–January and March [71,72]	Primary human-biting species in California [71]
*Ixodes holocyclus* (paralysis/scrub typhus tick)	Found along the East Coast of Australia, from northern Queensland down to Victoria [73]	The limited distribution is thought to be driven by climatic conditions, particularly humidity, and it is often found in wet forested (scrub) areas [73]	Generalist feeder, has been recorded infesting mammals and birds [74]. In south-eastern Queensland, northern brown bandicoots (*Isoodon macrourus*) and long-nosed bandicoots (*Perameles nasuta*) are required for *I. holocyclus* populations to persist from one season to the next [74]	Nymphs most abundant between April and September; female adults peak between October and December [74]	Known to frequently bite humans [75]
*Ixodes hexagonus* (hedgehog tick)	Widespread distribution across temperate Europe and North Africa [34]	Nidicolous, found in dark humid places e.g., nests/burrows [76,77]; distribution is not limited by or dependent upon ambient climatic conditions	Hedgehogs are the most common hosts, but also found on a range of mammals [34]	Infestations occur throughout the year [78,79,80,81]	Human biting has been reported [17,82,83,84,85,86]
*Amblyomma cajennense* s.l.	Range spans from parts of southern USA, across Central America, the Caribbean, extending into South America to parts of northern Argentina [87]	Found in a wide range of habitats including dry grasslands, tropical forests and highlands of the Peruvian Andes [87]	Giant anteaters, wild pigs, tapir, water buffalo, dogs, horses, capybara and small mammals are common hosts [88,89,90,91,92,93]	Larvae most active between August and February, peak nymph activity between December and March, adult activity increases when daily mean temperature reaches 20 °C, peaking between February and September [89,90,94,95,96,97]	All life stages bite humans, and in South America it bites humans almost twice as frequently as all other tick species combined [98,99]
*Hyalomma truncatum*	Widely distributed throughout sub-Saharan Africa	Unknown	Parasitises a wide range of mammals, including rodents, lagomorphs, canids, ungulates and carnivores [9,100,101]	Unknown	Human infestation is rare [102], but there are at least two reports of this tick species found attached to returned travellers [7,9]
*Rhipicephalus gertrudae*	Found extensively across South Africa, with its distribution reaching the southern province of Western Cape, as well as northwards into Namibia and eastwards to the Free State province [103]	Unknown	Larvae and nymphs: small mammals [104,105] Adults: cattle, goats, sheep, horses, zebra, antelopes, primates, dogs, cats [22,106,107,108,109,110]	Highest numbers of biting ticks occur between September and February [103,107,111]	Adults most commonly bite humans [111]
*Rhipicephalus appendiculatus* (brown ear tick)	Widely distributed between southern Sudan and the east coast of South Africa, with a noticeable absence in the Horn of Africa [112]	Unable to survive on open plains, and predominantly found in woodland and savannah with good vegetation cover, but can disappear following overgrazing [111]	Larvae and nymphs: small antelopes and hares Adults: large ungulates including cattle, buffalo, wildebeest [113]	Larvae active late summer to winter (April–August) Nymphs active in winter and early spring (July to October) Adults active in rainy season (December to March) [113]	All stages are known to bite humans [111]
*Haemaphysalis hystricis*	Distributed from the eastern Himalayas to India, through the coasts of Vietnam and China to Taiwan and Japan, and further south including Thailand, Malaysia and Indonesia [114,115,116,117,118,119]	Unknown	Commonly infests many hosts including cats, dogs, Japanese marten, wild boar, wildcats [115,117,120,121]	Unknown	Occasionally bite humans
*Dermacentor auratus*	Widespread distribution across much of Asia, including Borneo, China, India, Java, Laos, Malaysia, Myanmar, Nepal, Sri Lanka, Sumatra, Thailand and Vietnam [39,122,123,124,125,126,127,128,129,130,131,132]	It is a common tick species found in forests below 400 m altitude, although there are data of tick bites occurring at higher altitudes [131]	Larvae infest rodents; adults found on wild pigs, cattle, buffalo, deer, dogs and birds [125,126,129,131,132,133]	Unknown	Intra-aural infestation in particular has been reported [122,125,126,128,129,131,134]
